# Detection of mismatch repair gene germline mutation carrier among Chinese population with colorectal cancer

**DOI:** 10.1186/1471-2407-8-44

**Published:** 2008-02-07

**Authors:** Hei-Ying Jin, Xiufang Liu, Vicky Ka Ming Li, Yijiang Ding, Bolin Yang, Jianxiang Geng, Rensheng Lai, Shuqing Ding, Min Ni, Ronghua Zhao

**Affiliations:** 1 National Center of Colorectal Surgery, the 3^rd ^Affiliated Hospital of Nanjing University of Traditional Chinese Medicine. 1 Jinling Road, Nanjing 210001, China; 2 Department of Colorectal Surgery, the 1^st ^Affiliated Hospital of Nanjing University of Traditional Chinese Medicine. Hanzhong Road, Nanjing 210028, China; 3 Department of Surgery, Kwong Wah Hospital, 25 Waterloo Road, Kwoloon, HKSAR, China; 4 GI Genetic Research Lab, Cancer Center of University of Puerto Rico, Medical Science Campus of UPR, San Juan 00935, Puerto Rico

## Abstract

**Background:**

Hereditary nonpolyposis colorectal cancer (HNPCC) is an autosomal dominant syndrome. The National Cancer Institute (NCI) has recommended the Revised Bethesda guidelines for screening HNPCC. There has been a great deal of research on the value of these tests in other countries. However, literature about the Chinese population is scarce. Our objective is to detect and study microsatellite instability (MSI) and mismatch repair (MMR) gene germline mutation carriers among a Chinese population with colorectal cancer.

**Methods:**

In 146 prospectively recruited consecutive patients with clinically proven colorectal cancer, MSI carriers were identified by analysis of tumor tissue using multiplex fluorescence polymerase chain reaction (PCR) using the NCI recommended panel and classified into microsatellite instability-low (MSI-L), microsatellite instability-high (MSI-H) and microsatellite stable (MSS) groups. Immunohistochemical staining for MSH2, MSH6 and MLH1 on tissue microarrays (TMAs) was performed, and methylation of the MLH1 promoter was analyzed by quantitative methylation specific PCR (MSP). Germline mutation analysis of blood samples was performed for MSH2, MSH6 and MLH1 genes.

**Results:**

Thirty-four out of the 146 colorectal cancers (CRCs, 23.2%) were MSI, including 19 MSI-H CRCs and 15 MSI-L CRCS. Negative staining for MSH2 was found in 8 CRCs, negative staining for MSH6 was found in 6 CRCs. One MSI-H CRC was negative for both MSH6 and MSH2. Seventeen CRCs stained negatively for MLH1. MLH1 promoter methylation was determined in 34 MSI CRCs. Hypermethylation of the MLH1 promoter occurred in 14 (73.7%) out of 19 MSI-H CRCs and 5 (33.3%) out of 15 MSI-L CRCs. Among the 34 MSI carriers and one MSS CRC with MLH1 negative staining, 8 had a MMR gene germline mutation, which accounted for 23.5% of all MSI colorectal cancers and 5.5% of all the colorectal cancers. Five patients harbored MSH2 germline mutations, and three patients harbored MSH6 germline mutations. None of the patients had an MLH1 mutation. Mutations were commonly located in exon 7 and 12 of MSH2 and exon 5 of MSH6. Right colonic lesions and mucinous carcinoma were not common in MSI carriers.

**Conclusion:**

Our data may imply that the characteristics of HNPCC in the Chinese population are probably different from those of Western countries. Application of NCI recommended criteria may not be effective enough to identify Chinese HNPCC families. Further studies are necessary to echo or refute our results so as to make the NCI recommendation more universally applicable.

## Background

HNPCC is an autosomal dominant syndrome, which accounts for about 1–5% of colorectal cancer [1]. HNPCC patients are characterized by earlier symptoms, more mucinous carcinoma, more synchronous and metachronous colorectal tumors and more extra-colonic tumors, but have better survival [1,2]. It is believed that HNPCC is secondary to a germline mutation resulting in a defective MMR gene. A defective MMR gene results in increased DNA replication errors and MSI, which causes the occurrence of tumors in different organs, especially in the colorectum. Identification of HPNCC families is important because the diagnosis, treatment and follow up of these individuals should be different from those with sporadic colorectal cancer [2]. However, the clinical diagnosis of HNPCC patients is very difficult for lack of specific clinical phenotype. Though Amsterdam criteria I and II were established for HNPCC diagnosis[3,4], many HNPCC families still do not meet the criteria.

MSI is an important phenotype of MMR gene mutation. In 1997, the National Cancer Institute (NCI) recommended screening MSI CRCs using the Bethesda guidelines [5]. After compiling evidence from years of global studies and follow up, NCI revised the Bethesda guidelines in 2004, which is called the revised Bethesda standard [6]. NCI recommended screening of HNPCC based on detection of MSI in the tumor and loss of expression of a MMR gene using immunohistochemistry (IHC) staining [6]. However, until now, there has been no research about the applicability of the NCI recommendations to the Chinese population with colorectal cancers. The aim of this study is to detect and study MSI carrier and mismatch repair (MMR) gene germline mutation carriers among a Chinese population with colorectal cancer.

## Methods

### Patients

Data were prospectively collected from 158 consecutive colorectal cancer patients who received surgical treatments in the National Colorectal Center of the Third Affiliated Hospital of Nanjing University of Traditional Chinese Medicine (NUTCM) from October 2004 to June 2006. All patients signed an informed consent before the study. Patients who had histologically proven carcinoma and received operative treatment in our hospital were included in the study. Patients were excluded if they: (1) had evidence of concomitant ulcerative colitis, (2) presented with clinically unresectable diseases, (3) were diagnosed with FAP and other polyposis syndromes, (4) refused operative treatment or refused to participate in the study. This study was approved by the ethical committee of the NUTCM.

### Extraction of genomic DNA

Fresh tissue samples of tumor and matched normal mucosa were obtained from the surgical specimen once it was removed. DNA was extracted using a tissue DNA extraction kit from the Beijing Bio-lab Materials Institute (Beijing, China) and was stored at -80°C until analysis.

### Synthesis of fluorescent primer

A reference panel of 5 MSI markers recommended by NCI: BAT-26, BAT-25, D2S123, D5S346 and D17S250 were used in this study. The primers of BAT-40 and MYCL were also applied. The fluorescent primers were synthesized at Applied Biosystem Company (Foster City, CA) with a previously published method [6,7].

### Microsatellite instability analysis

Microsatellite instability was analyzed according to a method previously reported [6,7]. Briefly, the 5 microsatellites were amplified by multiple PCR. The reaction system consisted of 1 μL of template (extracted genomic DNA, 100 ng), 4 μL of mixed primers (primer mix) and 15 μL of ABI Prism True Allele PCR Premix (containing AmpliTaq Gold DNA Polymerase buffer, magnesium chloride and dNTPs) (Applied Biosystems Company). The reaction initially underwent pre-denature at 95°C for 15 minutes, then 30 cycles of: denaturation at 94°C for 1 minute, annealing at 56°C for 1 minute and extension at 72°C for 1 minute were performed. An additional extension at 72°C for 25 minutes was performed subsequently.

PCR reaction product (1 μL) was then mixed with 0.4 μL LIZ (internal standard) and 9 μL of formamide. Heat denaturation was performed on the mixture at 95°C for 5 minutes, and the sample was kept at 4°C for 5 minutes. Then the product was put in a 96 well plate, and capillary electrophoresis was performed with an AB13100-Avant sequencer (Applied Biosystem Shanghai Division, Shanghai, China) for 45 minutes. Data was automatically analyzed with Genotyper 2.5 software (Applied Biosystem Shanghai Division) and the original data was generated. The electrophoresis was then repeated on the next day to ensure the accuracy of the analysis.

The DNA sequence of each microsatellite in the tumor was compared with that of the matched normal mucosa. It was considered as unstable if there was an absence, shortening or prolongation of the DNA sequence. MSI-L was defined as 1 unstable microsatellite out of 5, while MSI-H was defined as more than 1 unstable microsatellite out of 5. If the tumor microsatellites were identical to those of the normal tissue, the microsatellite was considered as stable (MSS).

For the MSI-L CRCs, BAT-40 and MYCL were detected. If either of these two markers was unstable, this colorectal sample was considered as MSI-H.

### Immunohistochemical staining for MSH2, MSH6 and MLH1 on TMAs

The CRC microarray was constructed as previously described [8,9]. Briefly, formalin-fixed paraffin-embedded tissue blocks of CRC resections were cut from the donor block and stained with hematoxylin-eosin (HE). These slides were used to guide the sampling from morphologically representative regions of the tissues. A tissue array instrument (Beecher Instruments, Silver Spring, MD) was used to create holes in a recipient paraffin block and to acquire tissue cores from the donor block by a thin-walled needle with an inner diameter of 1.0 mm, held in an X-Y precision guide. The cylindrical samples were retrieved from the selected regions in the donors and extruded directly into the recipient blocks with defined array coordinates. Three cores were obtained from each sample. After the construction of the array block, multiple 4-mm thick sections were cut with a microtome using an adhesive-coated tape sectioning system (Instrumedics, Hackensack, NJ). In our analysis the rates of lost cases attributable to tissue damage were less than 5% for the different markers.

TMA slides were stained with MLH1 antibodies (1:50 dilution, No. sc-494, Santa Cruz, Biotrade Ltd, Shanghai, China) and MSH2 (1:100 dilution, No. sc-581, Santa Cruz, Biotrade Ltd, Shanghai, China). IHC staining for samples on the tissue microarray was carried out using Envision ready-to-use methods (Dako Diagnostics, Zug, Switzerland). Slides were deparaffinized in xylene and rehydrated through graded concentrations of ethanol to distilled water, and endogenous peroxidase activity was blocked by incubation with 30 mL/L H_2_O_2 _in methanol for 10 min at room temperature. Then sections were submitted to antigen retrieval in a pressure cooker containing 0.01 mmol/L natrium citricium buffer for 10 min. Slides were subsequently incubated in 100 mL/L normal goat serum for 20 min at room temperature. Sections were permeabilized in PBS-Triton and incubated overnight with primary antibody at 4°C. The pathologist and technician who reviewed the immunostaining of the tissue samples were blinded to the patient's information. Stained slides and individual cores were scored as either positive (showing nuclear staining in at least some tumor cells) or negative.

### Detection of methylation of MLH1 promoter by quantitative MSP

DNA was chemically modified by sodium bisulfite to convert all unmethylated cytosines to uracils, while leaving methylcytosines unaltered (EZ DNA methylation gold kit; Zymo Research, Orange, CA), and eluted in 50 μL of elution buffer [10,11]. The bisulfite-modified DNA was then used as a template for the fluorescence-based real-time PCR assay [12].

The sequences of primers and the fluorogenic probe were designed by MethPrimer software. Primer sequence for hMLH1 was: CGTTATATATCGTTCGTAGTATTCGTGTTT(Forward), and CTATCGCCGCCTCATCGT (Reverse), probe sequence was 6FAM-CGCGACGTCAAACGCCACTACG-TAMRA. For the MSP, 5 μL of bisulfite-converted DNA was used in each amplification. PCR was performed in a reaction volume of 25 μL consisting of 5 pmol of each primer, 250 pmol of probe, 200 μM each of dATP, dCTP, and dGTP, 400 μM dUTP, 3.5 mM MgCl2, 1× TaqMan Buffer A, and 2 units of AmpliTaq Gold polymerase (Applied Biosystem Shanghai Division, Shanghai, China) at the following condition: 95°C for 10 min, followed by 50 cycles at 95°C for 15 s, and 60°C for 1 min. All PCR was performed in the ABI-7000 Real-Time PCR Detection system (Applied Biosystem Shanghai Division, Shanghai, China). CpGenomeTM Universal methylated DNA (Chemicon International Inc., city, CA, USA) was included as positive control in all amplifications and glyceraldehyde-3-phosphate dehydrogenase (GAPDH) quantification of all untreated DNA was used as loading control.

### Extraction of genomic DNA from blood

Blood from peripheral veins (2 mL) was taken from patients with MSI colorectal cancer, and genomic DNA was extracted using a kit from Beijing Bio-Lab Materials Institute. The extracted genomic DNA was stored at -80°C until further analysis.

### Mutation of MSH2, MLH1 and MSH6 genes

Primers for all exons of MSH2, MLH1 and MSH6 were designed for PCR amplification as previously reported. PCR amplification was performed using the reagents from ABI Company, following the protocol provided by the company. After amplification, the PCR products were purified by electrophoresis through a 1.5% low melting point agarose gel, and then were sequenced on an AB13100-Avant sequencer (Applied Biosystem Shanghai Division) using fluorescently labeled primers, following the protocols supplied by the manufacturer. By comparing the obtained sequence with the known sequence, nonsense, missense, and frameshift mutations were identified. Nonsense and frameshift mutations were considered pathogenic. All missense mutations were screened in 50 patients with MSS colorectal cancer and 50 people without cancer or a family history of cancer. They were judged as pathogenic if they could not be found in MSS patients and normal people, otherwise, they were judged as polymorphisms.

### Statistical methods

The data are presented as mean and standard deviation (x ± SD) or percentage. Student t-test, chi-square test or Log-rank test were used for statistic analysis as appropriate (SPSS Inc., Chicago, IL, USA), with p < 0.05 indicating statistical significance.

## Results

One hundred fifty-eight patients received treatment for colorectal cancer in our Colorectal Center from October 2004 to June 2006. Three patients were excluded for concomitant ulcerative colitis and two for unresectable diseases. Five patients refused operation and 2 patients refused to participate in the study. 146 patients (84 male and 62 female, age: 60.8 ± 10.5 yr) were recruited into the study. Clinicopathological features of those patients are summarized in Table 1.

**Table 1:** Clinicopathological features of cases (N = 146)

***Cases***	***Amount***
Age	60.8 ± 10.5 yr
Gender:	
male	84(57.5%)
female	62(42.5%)
Location:	
ascending colon	24 (16.3%)
transverse colon	15 (10.3%)
descending colon	13 (8.9%)
sigmoid colon	16 (11.0%)
rectum	78 (53.4%)
Multiple cancer:	
synchronous tumours	3(2%)
metachronous tumour	7 (4.5%)
Pathology:	
adenocarcinoma	93 (63.7%)
mucinous carcinoma	23 (15.8%)
mixed type	30 (20.5%)
Dukes stage*:	
I	17 (11.6%)
II	69 (47.2%)
III	48(32.9%)
IV	12 (8.2%)
Family history	7
Amsterdam criteria	1

*Astler-Coller Dukes stage

### The results of the MSI analysis, hypermethylation of MLH1 and IHC staining of MSH2, MSH6 and MLH1

With the original NCI five marker panel, 34 of 146 patients (23.2%) were MSI, 17 were MSI-H and another 17 were MSI-L. Two out of 17 MSI-L CRCs were unstable with BAT-40 and were judged as MSI-H. Seven out of 17 MSI-H CRCs were unstable with BAT-40. In all, 19 CRCs (55.9%) were MSI-H and 15 cancers (44.1%) were MSI-L. The remaining 112 patients (76.8% were MSS. In the MSI group, 13 patients (8.9%) were BAT-26 unstable, 18 (12.3%) were BAT-25 unstable, 16 (11.0%) were D2S123 unstable, 11 (7.5%) were D5S346 unstable, 14 (9.6%) were D17S250 unstable.

Negative staining for MSH2 was found in 8 CRCs, negative staining for MSH6 was found in 6 CRCs. One MSI-H CRC was negative for both MSH6 and MSH2. Seventeen CRCs stained negatively for MLH1. Negative staining was not found in three MSI CRCs, and one CRC was found with negative MLH1 staining, but it was MSS.

MLH1 promoter methylation was determined in 34 MSI CRCs. Hypermethylation of the MLH1 promoter occurred in 14 (73.7%) out of 19 MSI-H CRCs and 5 (33.3%) out 15 MSI-L CRCs. The results of MSI and IHC staining are summarized in Table 2.

**Table 2:** The result of MSI, hypermethylation of MLH1 and IHC staining

IHC(-)	MSH2	MSH6	MLH1	None	Hypermethylation of MLH1
MSI-H	8*	4*	7	1	14
MSI-L	1	3	9	2	5
MSS	0	0	1	111	n.a.#

*One MSI-H CRCs was negative both for MSH6 and MSH2

# MLH1 promoter methylation was determined only in MSI CRCs.

### Comparison of clinical features between MSI and MSS colorectal cancers

Table 3 summarizes the clinical features of MSS and MSI patients. Patients in the MSS group are younger than those in MSI group (70.9 ± 17.8 versus 61.0 ± 9.4, p = 0.045). Twenty out of 34 (59%) MSI patients were stage III or stage IV, significantly higher than those (35.7%) in the MSS group (p = 0.008). There were no differences in other demographic and clinical pathological features, including sex, tumor location and type between the two groups. The most common location of the tumor was the rectum in both groups, accounting for more than 50% of the cases. Adenocarcinoma was the most common pathological type in both groups. Ten patients in the MSI group (29.4%) had mucinous carcinoma.

**Table 3:** Comparisons of clinical features between MSS and MSI group

	**MSS (n = 112)**	**MSI (n = 34)**		**P-value**
			
		**MSI-L (n = 15)**	**MSI-H (n = 19)**	
Mean age (range)	60.0 (26–84)	71.9 ± 17.8	60.4 ± 9.4	0.010
Gender				
Male	67	8	12	0.207
Female	45	7	7	
Location of tumor				0.658
Ascending colon	19	1	4	
Transverse colon	9	1	4	
Descending colon	10	2	0	
Sigmoid flexure	11	1	4	
Rectum	61	10	7	
Pathological type				0.545
Adenocarcinoma	71	11	13	
Mucinous cancer	39	4	6	
Mixture type	2	0	0	
Dukes' Staging#				0.008
Stage I	15	2	0	
Stage II	57	5	7	
Stage III	34	6	8	
Stage IV	6	2	4	
Degree of differentiation				0.169
Well	35	6	5	
Moderate	58	3	6	
Poor	11	6	8	

*Statistical analysis was made between MSI and MSS group only

### MSI state, germline mutations, and consequential changes in amino-acid sequence

The MSI state, mutation sites, and consequential changes in amino-acid sequences of MMR genes are summarized in Table 4. In 34 MSI CRCs and one MSS CRC with MLH1 negative staining, 8 had MMR gene germline mutations, accounting for 22.9% of MSI colorectal cancers and 5.5% of all colorectal cancers. Three patients had MSH6 germline mutations and 5 had MSH2 germline mutations; the clinical features of these patients are summarized in Table 5. None of the patients had MLH1 gene mutations. Seven patients in the MSI group had A/T heterozygosis in MSH6 codon 380 of exon 5, but it did not cause changes in the amino acid sequence. The germline mutations of the MSI-L and MSI-H CRCs are summarized in Table 6. Six CRCs with mutations were MSI-H and two patients with mutations were MSI-L.

**Table 4:** Details of the 8 patients in MSS group identified to have MMR gene germline mutation

**Patient No.**	**MSI state**	**Mutant gene**	**Exon**	**Mutation site**	**Change in amino acid sequence**
2	L	MSH2	12	c.1886 A > G	p.629 Gln > Arg
28	H	MSH2	7	c.1225 C > A	p.409 Gln > Lys
93	L	MSH6	5	c.3200 C > A	p.1067 Pro > His
98	H	MSH2	12	c.1886 A > G	p.629 Gln > Arg
115	H	MSH6	2	c.440 T > A	p.149 Leu > His
119	H	MSH2	7	c.1145 G > A	p.382 Arg > His
132	H	MSH6	5	c.3185 C > T	p.1062 Pro > Leu
151	H	MSH2	7	c.1168 C > T	p.390 Phe > Leu

**Table 5:** Clinical features of patients in MSI group with MMR gene mutations

**Patient No.**	**Age**	**Sex**	**Tumor position**	**Pathology**	**Dukes' stage**	**MSI state**	**Family history**	**Multiple primary**
2	49	F	Sigmoid	Adenocarcinoma	C1	H	No	No
28	54	F	Rectum	Adenocarcinoma	B1	H	No	No
93	68	M	Rectum	Adenocarcinoma	B2	L	No	No
98	78	F	Sigmoid	Mucinous carcinoma	B1	H	No	No
115	57	F	Rectum	Adenocarcinoma	C2	L	No	No
119	61	F	Rectum	Adenocarcinoma	B1	H	No	No
124	34	F	Hepatic Flexure	Mucinous carcinoma	C2	H	Yes*	No
151	68	F	Rectum	Adenocarcinoma	C1	H	No	No

*Amsterdam criteria II

**Table 6:** The mutation between the MSI-L and MSI-H

	**N**	**Mutation**		
		
		**total**	**MSH2**	**MSH6**
MSI-L	17	2	1	1
MSL-H	17	6	4	2
total	34	8	5	3

P = 0.112

### Clinical features of patients in the MSI group with MMR gene mutations

The clinical features of MSI patients with MMR gene mutations are shown in Table 5. Mean age was 58.8 years (range: 34–78). Six were female and 2 were male. Only one patient had right side colonic lesion and 2 had mucinous carcinoma. There were no patients in Dukes' A or with synchronous/metachronous disease. Only one patient fulfilled the Amsterdam Criteria (II).

## Discussion

Identification of mismatch repair gene germline mutations in sporadic colorectal cancer is the most important method to screen HNPCC. In 2004, the NCI recommended the revised Bethesda criteria as a HNPCC screening guideline and established the HNPCC diagnostic procedures. These guidelines stated that MSI should firstly be identified in colorectal tumor tissue, and then genetic tests would be performed to confirm MMR gene germline mutations in a blood sample [6].

At present, a number of publications have studied the value of the revised Bethesda criteria and the relevant diagnostic procedures [13,14]. However, there has not been any systematic report on germline mutations of MMR genes in the Chinese population. Moreover, we found that research from some countries have results that, if applied to the NCI recommendations, may have resulted in a missed HNPCC family or low pick-up rate of HNPCC. In a study by Pinol et al. from Spain [13], 287 out of 1222 patients (23.5%) complied with the revised Bethesda standard. Ninety-one patients (7.4%) were MSI carriers, but only 11 patients (0.9%) had germline mutations of MSH2 or MLH1. This means that among this group, only 0.9% of patients could be diagnosed as HNPCC. In a study by Yearsley et al. [14], out of 482 US patients with colorectal cancers, 87 patients (18%) were MSI carriers and only 12 cases (2.5%) had MMR gene germline mutations. These results may be explained by different case selections and sensitivity of the tests used. On the other hand, in their study of HNPCC related tumors in young patients (<50 years old), Niessen et al. [15] found that the rate of MSH2, MSH6 and MLH1 germline mutations in MSI carriers was 82%. In our 146 patients with colorectal cancers, 34 patients (23.3%) had MSI colorectal cancer. This is comparable to the studies by Pinol et al. [13] and Yearsley et al. [16]. Wong et al. [17] showed that in the case of sporadic endometrial carcinoma, MSI endometrial carcinoma accounted for 26% of patients. Our results showed that there were 15 (10.3%) CRCs with MSI-L and 19 (13.0%) CRCs with MSL-H, respectively. Lamberti et al. [18] reported that MSI-L and MSI-H accounted for 6% and 17%, respectively, of German patients. These results are similar to ours. Unexpectedly, in the present study, the age of patients with MSS colorectal cancers (60 yrs) was similar to the age of patients with MSI-H (61 yrs). In contrast, the average age of patients with MSI-L colorectal cancer was 71, which was quite different from the reports of other research groups [13,15,18]. In a study of 1263 patients with colorectal cancers, Benatti et al. [19] found that those who were MSI carriers tended to have mucinous and right colonic tumours. Noda et al. [20] also found that MSI carriers have more right colonic tumors. However, in our study, tumors in the MSI group were most commonly located at the rectum, and mucinous carcinoma was not the most common pathological type, as it only accounted for 29%. Interestingly, patients in our MSI-L group were older than the MSS group. The reason for these findings is uncertain. Bettstetter et al. [20] reported that the average age of MSI CRCs with MLH1 negative staining was 80 yrs, which was similar to our results. Most of these MSI CRCs were caused by MLH1 promoter hypermethylation. In our group, out of 34 MSI CRCs, 19 CRCs (55.9%) were hypermethylated at the MLH1 promoter, which accounted for 73.7% of MSI-H CRCs and 33.3% of MSI CRCs. Anacleto et al. [21] reported that 8 out of 15 MSI CRCs had MLH1 promoter hypermethylation, which was similar to our results. Kim et al. [10] found that in MSI-H gastric cancers, the MLH1 hypermethylation occurred in 89% of patients. Bettstetter et al. [20] showed that all sporadic MSI-H CRCs were hypermethylated at the MLH1 promoter. These results were similar to ours. Fourteen out of 16 MLH1 negatively staining CRCs were hypermethylated at the MLH1promoter. Mutation analysis revealed that 8 patients (23.5%) had MMR gene germline mutations out of 34 MSI patients. Five patients had MSH2 mutations and 3 had MSH6 mutations, while no MLH1 mutation was found. There were 2 and 6 patients who had mutations in MSH2 and MSH6 in the MSI-L and MSI-H groups, respectively. Yearsley et al. [15], in their study of 87 patients with MSI colorectal cancers, found 12 patients (13.8%) with MLH1 and MSH2 germline mutations. Niessen et al. [16], however, found that the rate of MSH2, MSH6 and MLH1 germline mutations was 82% in the young age group (<50 years old). This was quite different from our group. This difference might be due to different case selections of the two groups. Our study was more representative of the patient population because it was a successive cohort study. Most importantly, the above differences may also be explained by underlying differences in genetic background between Chinese and Western populations.

Regarding the location of gene mutations, we found that 3 patients bore mutations in exon 7 of MSH2, 2 had mutations in exon 12 of MSH2, 1 had a mutation in exon 2 of MSH6, and 2 had mutations in exon 5 of MSH6. No MLH1 mutations were found in our cohort. All 8 mutations were considered pathogenic mutations. The mutation of C1886 A > G, C1668 C > T was reported as a pathogenic mutation previously [22-25]. However, the other six mutations have not been reported in other publications and are likely to be novel mutations. We also attempted to screen for the presence of all these mutations in 50 randomly selected patients in the MSS group and in 50 normal people. The result of this screen was negative, and all these CRCs with mutations stained negatively for MSH2 or/and MSH6, indicating that these 6 mutations may be pathogenic mutations but not polymorphisms. In terms of the clinical features of the 8 cases with mutations, only 1 conformed to the Amsterdam criteria II; all the others did not have any family history of malignancy. Moreover, the median age was 59 with 4 patients that were older than 60 years old.

One limitation of this study is that it is a single-center study with a relatively small sample size. Therefore, the results of this study need to be confirmed by a well designed multi-center study, which is one of our ongoing studies in China.

## Conclusion

Our results imply that HNPCC in the Chinese population may have distinct clinicopathological characteristics and underlying MMR germline mutations, as compared to patients from Western countries. Application of NCI recommendations on the Chinese population may not enable the screening of all HNPCC families. Further studies are necessary to echo or refute our results so as to make the NCI recommendation more universally applicable.

## Competing interests

The author(s) declare that they have no competing interests.

## Authors' contributions

HYJ designed the study and wrote the paper. XFL carried out the molecular diagnosis. VKML carried out sample collection and draft the manuscript. YD participated in the design of the study and data analysis. BLY, JXG and RSL carried out immunoassay. SQD participated in the data analysis. MN established TMA and data analysis. RHZ participated in the design of the study and data analysis. All authors read and approved the final manuscript

## Pre-publication history

The pre-publication history for this paper can be accessed here:


